# Prevalence of Maternal Cytomegalovirus Antibodies and Neonatal Congenital Cytomegalovirus at Less than 34 Weeks of Gestation: A Prospective Study

**DOI:** 10.1055/s-0042-1756641

**Published:** 2022-10-19

**Authors:** Shilpa U. Kalane, Lavannya Raste, Sampada Patwardhan, Digna A. Beasley, Uday P. Devaskar

**Affiliations:** 1 Department of Neonatology, Deenanath Mangeshkar Hospital, Pune, Maharashtra, India; 2 Department of NICU Nutritionist, Deenanath Mangeshkar Hospital, Pune, Maharashtra, India; 3 Department of Microbiology, Deenanath Mangeshkar Hospital, Pune, Maharashtra, India; 4 Department of NICU, Neonatology, Centinela Hospital, Inglewood, California; 5 Department of Pediatrics, Neonatology, David Geffen School of Medicine at University of California Los Angeles, Los Angeles, California

**Keywords:** maternal CMV-IgG antibody, premature neonate, congenital cytomegalovirus

## Abstract

**Objective** Congenital cytomegalovirus (cCMV) acquired postnatally can lead to hearing loss and adverse central nervous system (CNS) function, especially in the preterm neonate. We prospectively determined the prevalence of maternal serum CMV-immunoglobulin (IgG) and the incidence of cCMV at <34 weeks of gestation.

**Study Design** Study was conducted in the United States and India. Maternal blood was collected within 5 days after delivery. CMV-IgG antibodies were quantitated by an immunoassay. Baby's urine at birth was tested for CMV-DNA by the polymerase chain reaction.

**Results** In total, 65 women and 74 neonates were studied. In the United States, 6 out of 21 (76%), while in India, 42 out of 44 (96%) mothers were seropositive (combined 89%). In the United States, none of the neonates had CMV in the urine, while in India 4 out of 52 (7.7%) were positive (combined 5.4%)

**Conclusion** Mother's blood and baby's urine should be tested for serum CMV-IgG antibodies and CMV-DNA at delivery at <34-weeks of gestational age. Targeted screening will help in making an early diagnosis of cCMV, initiate therapy, and detect and treat early CNS problems including hearing loss.

**Key Points**

Maternal serum CMV screening after premature delivery at less than 34 weeks of gestation.

Neonatal urine CMV screening at less than 34 weeks of gestation.

Prematurity: importance of CMV during premature labor and delivery at less than 34 weeks.

Cytomegalovirus (CMV) is a ubiquitous virus infecting approximately 85% of people of all ages worldwide. 1 The prevalence of CMV depends on the national and regional geography, ethnicity, social customs, and low socioeconomic status. 2 In pregnancy, CMV can lead to stillbirth or intrauterine growth restriction. 3 Congenital CMV (cCMV) is an important public health problem among pediatric patients. 4 In children, the spectrum is wide ranging from asymptomatic disease in the healthy full-term newborn to death in the immune-comprised host such as very preterm (VPT) infants. 4 cCMV is a frequently occurring but underrecognized global health problem described as “an elephant in our room”. 5 In full-term neonates, CMV is the most common infection with a universal prevalence of 0.23 to 0.64%, 6 7 8 while in the preterm, it is 1.3 to 2.3%. 9 10 In the United States alone, approximately 40,000 infants a year are born with cCMV, approximately 400 (1%) die from CMV and approximately 8,000 (25%) develop long-term permanent outcomes ranging from cognitive or motor disabilities to sensorineural deafness. 11 cCMV is the leading nongenetic etiology of sensorineural deafness and developmental sequelae during childhood. 12 In 10 to 20% of term infants who are asymptomatic at birth, neurologic deficits diagnosed later in life can be attributed to CMV. 3 13 14 15 16

CMV can be transmitted placentally, via aspiration of secretions in the birth canal or exposure to infected secretions such as saliva or breast milk (BM). 17 At birth, most CMV infections are asymptomatic and relatively innocuous in immunocompetent full-term neonates. 4 5 It was initially described as a form of natural immunization associated with minimal or absent signs of disease. 18 19 However, CMV acquired in utero or postnatally from mother's breast milk (MBM) can be problematic, especially in VPT neonates. 1 14 17 20 21 22 23 24 25 cCMV or acquired CMV is known to cause progressive hearing loss, and very low birth weight infants are known to be at a higher risk. 20 21 22 23 24 25 In a recent study, postnatal CMV infection acquired from MBM in VPT neonates was associated with adverse hearing and growth, and longer hospital stay. 20

Recently, we encountered a VPT neonate who developed septic shock due to CMV at 17 days of age after a relatively benign initial presentation. Review of her surprising clinical course motivated this study. It was undertaken to determine the incidence of the seropositive rate of CMV at the time of birth in women who had delivered infants at <34 weeks of gestational age (GA) and the prevalence of cCMV in these neonates. Here, we share our experience and lessons learned from first 65 mothers and their 74 infants.

## Index Case

A female infant with a GA of 24 ^1/7^ weeks and body weight of 660 g was born by vaginal route after spontaneous preterm labor of unknown etiology at Centinela Hospital (CH). There were no congenital anomalies. CH is a community hospital in Los Angeles area with a level III neonatal intensive care unit (NICU). At birth, she developed respiratory distress requiring supplemental oxygen, conventional mechanical ventilation, and exogenous surfactant. She was empirically treated with ampicillin and gentamycin for 2 days for possible bacterial sepsis. She was also treated with caffeine, donor breast milk (DBM), total parental nutrition and intralipids. She also received mother's expressed breast milk (EBM) and was treated with phototherapy for hyperbilirubinemia of prematurity. After 2 days of mechanical ventilation, the baby was extubated and transitioned to supplemental oxygen by nasal continuous positive airway pressure. By the seventh day of life, she was on 1 L per minute of nasal cannula oxygen and full feeds (DBM and MBM). On the 17th day of life, she clinically decompensated and developed signs and symptoms of septic shock. She required endotracheal intubation, mechanical ventilation with 100% oxygen, and vasopressors such as dopamine and epinephrine. She was treated with vancomycin and meropenem despite having negative blood culture, after obtaining blood culture which remained sterile. She was urgently transferred to Ronald Reagan-University of California at Los Angeles (UCLA) hospital for quaternary care. Upon arrival at UCLA for admission, she developed necrotizing enterocolitis with gastrointestinal perforation requiring an emergency laparotomy. While we did not check baby's urine for CMV at birth, it was positive at 20 days of age. Similarly, while we had not checked maternal serum at birth, it was positive for CMV-immunoglobin G (IgG) and IgM antibodies at 20 days postpartum. Mother's EBM at 20 days of age grew CMV. Infant was treated with IV ganciclovir and oral valganciclovir while monitoring for leucopenia and elevated liver enzymes. Infant was discharged home after a long NICU stay with chronic lung disease and irreversible CNS sequalae. Since we did not test her urine at birth, we could not determine with certainty if she was born with CMV that later flared versus CMV acquired postpartum from MBM.

## Materials and Methods

CH serves patients from a low socioeconomic class. It has approximately 700 deliveries a year and admits approximately 110 neonates in the NICU annually. All infants are in-born. In addition to CH, this study was also done at Deenanath Mangeshkar Hospital (DMH) (Pune, India). There is a very high rate (95%) of maternal seroprevalence for CMV in India. 18 26 DMH hospital is a tertiary care NICU and admits approximately 1,000 neonates a year (in and out-born) and has approximately 3,600 deliveries annually. The protocol was approved by the CH-Medical Board (identifier: NICU-PO13) and the Institutional Review Board (IRBs) at UCLA (identifier: 22-000107) and DMH (identifier: IHR-2021-Feb-UD-398). All mothers who delivered a neonate at <34 weeks of GA, irrespective of the etiology of prematurity, were approached for serum CMV-IgG antibody testing.

### Quantitation of Serum Cytomegalovirus Immunoglobin G Antibodies

Higher than normal concentration of serum CMV-IgG antibodies implies that the mother has current, past, or reactivated CMV infection. 27 28 Soon after birth, all mothers who had delivered infants <34 weeks of GA, irrespective of the etiology of prematurity, were approached. The rationale for testing their blood for CMV-IgG antibodies was explained, and verbal consent to draw approximately 2 mL of blood was obtained. At CH, quantitation of CMV immunoglobin G (IgG) antibodies was outsourced to LabCorp, Burlington, NC 28 and took approximately 3 days to get the results. It was a chemiluminescent microparticle immunoassay (test: 006494, CPT: 86644). Test results included negative: <0.6 aU/mL, equivocal: 0.6 to 1.0 aU/mL, and positive >1 aU/mL. Assay ranged from 0.6 to 10 aU/mL. 28

At DMH, maternal CMV-IgG antibody testing was performed in the clinical laboratory using an automated quantitative enzyme-linked fluorescent assay by the VIDAS device (Bio-Merieux, Marcy-l'Etoile, France). 29 Instructions by the manufacturer were strictly followed. It was a two-step sandwich immunoassay. Results were reported as arbitrary units (AU)/mL in comparison to the calibration curves stored in the instrument. The linearity of the assay was up to 400 AU/mL. The sensitivity and the specificity were 98.26 and 100%, respectively. Assay results included negative: <4 AU/mL, equivocal: 4 to 6 AU/mL, and positive: >6 AU/mL. 29 Positive or equivocal results were confirmed in duplicate.

### Urine for Cytomegalovirus

Bagged urine specimens were obtained from infants upon NICU admission. All samples were processed for the detection of CMV-DNA by polymerase chain reaction (PCR) technology. At CH, it is outsourced to LabCorp laboratory (PCR: 138693; CPT: 87496). 30 At DMH, it was tested by using the Gene Path qPCR diagnostic test kit. 31 Both are qualitative and quantitative assays.

### Statistical Analysis

All means and incidence were calculated.

## Results

At CH, 21 (all inborn) and at DMH 44 (36 inborn + 8 outborn) women were recruited. All mothers who delivered a neonate at <34 weeks of GA consented for serum IgG antibody testing. Maternal and neonatal data are shown in Table 1 . Three maternal blood samples were collected before imminent preterm delivery, while the rest were obtained before 5 days of baby's life. At CH, 16 out of 21 (approximately 76%) were seropositive (range 1.8– > 10 u/mL), while five (24%) were negative (<0.6 u/mL). In four mothers, IgG concentrations were >10 (upper limit of the assay). In one patient, the serum CMV-IgG level was 4.1 u/mL at 24 weeks of GA at another hospital and 3.1 at CH at the time of delivery at 28 weeks of GA. At DMH, 42 out of 44 (approximately 96%) were seropositive, while two were negative (approximately 4%). An average CMV-IgG concentration among seropositive women was 46 AU/mL (range: 11–105).

**Table 1:** Maternal and neonatal data (means, range, and percentage)

Characteristics	Centinela hospital (CH)	Deenanath Mangeshkar Hospital (DMH)	CH + DMH
Maternal age	25.3 (16–42) ( *n* = 21)	29.5 (19–43; *n* = 44)	28.5 (16–43)
Gravida	2.8 (1–7)	1.6 (1–6)	2.1 (1–7)
GA at delivery	31 ^2/7^ (25–33 ^4/7^ )	32 1/7 (26–34)	31 1/7 (25–34)
Vaginal vs. C-section	12/9	18/26	30/35
Maternal CMV-IgG (+ve)	16/21 (76%)	42/44 (95%)	58/65 (89%)
neonate gender (M/F)	12/10	32/22	44/32
neonate BW (g)	1,579 (660–2,550)	1,290 (630–1,900)	1,390 (630–2,550)
neonate RDS	13/22 (60%)	38/64 (70%)	51/74 (69 5)
neonate urine (CMV +ve)	0/22	4/52 (7.7%)	4/74 (5.4%)
neonate length of stay (d)	39 (6–98)	42 (0–105)	41 (0–105)

Abbreviations: BW, birth weight; CMV, cytomegalovirus; GA, gestational age; IgG, immunoglobulin; F, female; M, male; RDS, respiratory distress syndrome.

At CH, there were 22 (one twin), while at DMH, there were 52 neonates (six twins and one triplet). At DMH, two neonates died because of refractory respiratory distress secondary to extreme prematurity. At CH, none of the 22 neonates in this cohort had CMV in the urine, while at DMH, four babies were positive (three males and one female). The number of copies/mL was 47, 130, 185, and 5,024. Mothers of all four neonates with cCMV were seropositive for CMV-IgG antibodies. The combined rate of cCMV was 5.4% (4 out of 74). The rate of cCMV was 6.9% (4 out of 58) among all women who were seropositive for CMV-IgG antibodies. All four neonates born out of singleton pregnancy with cCMV did not have any signs or symptoms suggestive of active CMV disease. They will be followed closely during the NICU stay and infancy.

## Discussion

Maternal CMV infection can be diagnosed by testing IgM, low avidity IgG, IgG, or a combination of these antibodies. 3 27 32 IgM is an unreliable indicator for acute infection. 32 In addition, IgM can be frequently falsely positive (up to 90%) in patients with other viral infections or autoimmune diseases. 32 The anti-CMV IgG avidity test indicates the strength with which the antibody binds to the antigen. 3 32 Low avidity IgG is found only after acute infection and lasts for only 16 to 18 weeks. 3 32 After a person has CMV infection, IgG antibodies increase gradually and persist for the rest of an individual's life. 27 28 A positive test for CMV-IgG antibodies indicates that a person was infected with CMV at some time during his life but does not indicate when. 27 28 Thus, IgG testing is useful in distinguishing who has been exposed to the CMV from those who have not. 27 28 Higher than normal concentration of maternal serum CMV-IgG antibodies means that mother has current, past, or reactivated CMV infection. 27 28 While consideration was given to test for CMV-IgG avidity, for reasons described above, we chose to quantitate CMV-IgG antibodies only. 27 28 Mechanisms for wide variations in the maternal CMV-IgG levels among mothers in the United States and India are unknown. It could be due to recent versus old infection, reactivation of CMV, different CMV viral loads or virulence, ethnicity or socioeconomic status, difference in individual antibody response, or a combination of these factors.

It has been well established that urine PCR testing is highly reliable for the diagnosis of cCMV infection with a sensitivity of 100% and specificity of 99%. 33 34 One negative urine specimen excludes infection, and repeat sampling is not necessary. 34 After 21 days of age, positive urine for CMV could be due to CMV acquired postnatally from passage through the birth canal or MBM. As PCR techniques have become more sensitive, testing before 14 days is recommended to diagnose cCMV. 34

In this study, we simultaneously determined maternal CMV-IgG status and the presence of cCMV in neonates born at <34 weeks of GA. All neonates with CMV, congenital or postnatally acquired during the process of being born or transmitted via MBM, are born to women who are CMV-IgG seropositive. 2 35 If the mother is seronegative, her neonate will not have cCMV or develop it postnatally. 2 35 In our study, all neonates with cCMV were born to mothers who were seropositive. Even after our index patient developed septic shock at 17 days of age, diagnosis of CMV was not made for almost a week. If maternal CMV status at birth was known, we would have tested baby's urine for CMV at birth and periodically thereafter. In addition, we would have considered various options to eliminate CMV from the MBM. 2 17 We could have included CMV as an etiology for septic shock and initiated antiviral pharmacotherapy in a timely manner, perhaps leading to a better outcome. The lack of early diagnosis of CMV resulted in inappropriate antibiotic therapy, missed opportunity for early antiviral therapy, and subsequent development of chronic lung disease and neurologic disability.

CMV is reactivated in healthy immunocompetent seropositive women during pregnancy and lactation. 2 17 26 Maternal CMV seroprevalence rate in Western Europe, Canada, Australia, and the United States ranges from 40 to 60%, while it is >90% in South Africa, Brazil, India, Japan, and Turkey. 2 17 26 In our study, the maternal seroprevalence rate at CH was 76% which is higher than that reported previously. 2 17 26 It may be due to a low socioeconomic population. The seroprevalence rate in India was 96%, comparable to what has been reported. 2 17 26

Screening for CMV during pregnancy or at birth provides an opportunity to identify potentially presymptomatic neonates and consider an early intervention to ameliorate the morbidity. Selective maternal testing is performed in some parts of United States, Europe, Australia, and Israel. 36 37 38 39 The American Academy of Pediatrics (AAP) states that mothers who deliver infants at <32 weeks' of gestation can be screened for CMV. 40 However, CMV testing in these women is still not a standard practice. Results of our study further support the AAP statement. 40 Rather than screening all pregnant women for CMV, screening only those who have delivered premature infants should be acceptable and cost-effective. Nonetheless, local experience should guide in the development of a standard policy statement.

While practices vary widely among various NICUs, infant's urine is not routinely screened for CMV at birth or tested periodically thereafter to diagnose cCMV or acquired CMV. We propose that VPT infant's urine should be tested for CMV at birth and frequently thereafter especially if the mother is seropositive. However, if the mother is seronegative at birth, testing infant's urine at birth and thereafter may be considered for extremely preterm infants. This will allow for efficient differentiation between cCMV and postnatally acquired CMV. In addition, we will be able to identify neonates who are CMV positive but asymptomatic and need close follow-up during infancy.

If the mother is seronegative, even a preterm neonate can continue to receive her BM without concern for cCMV or acquired CMV. If the mother is seropositive, CMV is reactivated in the mammary gland in 96% of lactating women, and the virus is shed in BM in 80% (range: 70–95%). CMV reactivation during lactation is a local process restricted to the breast, with no signs of systemic spread. 41 42 Mechanisms of viral shedding exclusively in BM are not clear. In preterm neonates, the rate of MBM-acquired CMV infection varies from 6 to 60%. 17 It is GA dependent: 57% at 23 to 24 weeks, 17% at 25 to 26 weeks, 15% at 27 to 28 weeks, and 7% at 29 to 30 weeks. 17 Thus, if the mother is seropositive, preventing infection in VPT infants becomes paramount because morbidity and mortality can be severe with short- and long-term sequalae. 1 2 17 20 39 Various options to eliminate CMV in MBM need to be considered. These include freezing, short- or long-term pasteurization, and microwave or ultraviolet-c irradiation. 1 2 17 41 42

In full-term neonates, CMV infection is usually asymptomatic due to protection conferred by maternal IgG antibodies, passively acquired after approximately 28 weeks of gestation. VPT infants do not have this protection. Therefore, they are at the highest risk of developing severe postnatal CMV disease and long-term sequalae. 1 7 17 20 41 In term infants, the infection may be transmitted at the oropharynx or nasopharynx, while in preterm infants, it may be through the gastrointestinal mucosa. 1 21 41 Infants with cCMV infection may be asymptomatic or symptomatic at birth. If an infant is symptomatic, treatment with valganciclovir or ganciclovir is indicated. 17 41 The severity of long-term adverse outcomes varies substantially, from minimal deficits with unilateral sensorineural hearing loss to major neurodevelopmental complications and death. 4 6 7 8 Nearly 10 to 20% of initially asymptomatic CMV-infected neonates later developed hearing loss, 3 13 14 15 16 41 at which point the capacity for CMV diagnosis and opportunities for early interventions are lost or reduced. 41 Therefore, close follow-up including monitoring for hearing loss during infancy seems warranted. In addition, asymptomatic preterm babies, who have CMV in the urine during the immediate newborn period but are asymptomatic, could be treated with short-term course of oral valganciclovir. No data are available to support or refute this hypothesis.

## Conclusion

In summary, women who have delivered premature infants at <34 weeks of GA should be tested for the presence of serum CMV-IgG antibodies at the time of birth. This will allow us to make an early diagnosis of cCMV, consider various feeding options, and institute pharmacotherapy if indicated during the NICU stay. It will also guide us to detect and treat early neurodevelopmental disabilities including hearing loss.
